# Role of Met Axis in Head and Neck Cancer

**DOI:** 10.3390/cancers5041601

**Published:** 2013-11-26

**Authors:** Yiru Xu, Gary J. Fisher

**Affiliations:** Department of Dermatology, University of Michigan, Ann Arbor, MI 48109, USA

**Keywords:** head and neck cancer, receptor protein tyrosine kinase, hepatocyte growth factor receptor

## Abstract

Head and neck cancer is the sixth most common type of cancer worldwide. Despite advances in aggressive multidisciplinary treatments, the 5-year survival rate for this dreadful disease is only 50%, mostly due to high rate of recurrence and early involvement of regional lymph nodes and subsequent metastasis. Understanding the molecular mechanisms responsible for invasion and metastasis is one of the most pressing goals in the field of head and neck cancer. Met, also known as hepatocyte growth factor receptor (HGFR), is a member of the receptor protein tyrosine kinase (RPTK) family. There is compelling evidence that Met axis is dysregulated and plays important roles in tumorigenesis, progression, metastasis, angiogenesis, and drug resistance in head and neck cancer. We describe in this review current understanding of Met axis in head and neck cancer biology and development of therapeutic inhibitors targeting Met axis.

## 1. Introduction

Cancer from the head and neck region is the sixth most common cancer and accounts for some 350,000 cancer deaths worldwide each year [1,2]. More than 90% of head and neck cancer is squamous cell carcinoma (HNSCC) originated from the squamous epithelium lining of upper aerodigestive tract. The most prominent contributors for the development of HNSCC include tobacco use, alcohol consumption, and human papillomavirus (HPV) infections [3,4]. High mortality rates and severe morbidity are two common features of HNSCC. HNSCC affects tongue, oral cavity, oropharynx, hypopharynx, larynx, and nasopharynx and thus compromises essential functions such as speech and swallowing. Close to half of HNSCC patients will die from their disease within five years [5]. Despite advances in aggressive multidisciplinary treatments during the last two decades, the 5-year survival rate for HNSCC has not improved significantly and is lower than that for other major cancer types such as lymphoma, breast cancer, and malignant melanoma [6].

Traditional treatment for HNSCC has been extensive surgery, which often yields a poor functional outcome. In recent years, improved therapy has been developed involving intensity-modulated radiation and concurrent use of chemotherapy [7]. In addition, advances have been made in elucidating molecular mechanisms and tumor biology that are important for the development and progression of HNSCC. Evidence demonstrates the importance of epidermal growth factor receptor (EGFR) tyrosine kinase signaling pathways in tumorigenesis and progression of HNSCC. This information has led to application of therapeutic strategies that target EGFR. Indeed, cetuximab, a monoclonal anti-epidermal growth factor receptor (EGFR) antibody, has been shown to improve median survival, when combined with cytotoxic chemotherapy in treatment of advanced HNSCC [8]. Hepatocyte growth factor receptor (HGFR, also known as Met) pathway, is another important growth factor pathway that has been shown to be important for the development of HNSCC. A growing body of evidence suggests that Met pathway could function as a driving force for invasive growth and early metastasis of HNSCC, and may cooperate with EGFR to cause resistance against anti-EGFR therapy. Therefore, Met is an attractive therapeutic target for treatment of HNSCC [9]. 

## 2. The Met Signaling Pathway and Functions

Hepatocyte growth factor (HGF), also known as scatter factor, is active in numerous tissues [10]. HGF is synthesized and secreted by mesenchymal cells as a 90-kDa inactive single-chain polypeptide and then proteolytically cleaved into an active disulfide-linked heterodimer [11]. HGF participates in the regulation of organogenesis, tissue repair, angiogenesis and neural induction [11]. HGF is a unique growth factor that elicits multiple cellular responses. HGF is mitogenic in many normal cell types, including epithelial cells, vascular endothelial cells, and melanocytes. HGF can also function as a morphogen that induces transition of epithelial cells into a mesenchymal morphology and formation of branched tube-like structures [12,13]. HGF induces random movement/scattering in epithelial cells as well as dissociation, migration, and invasion of cells through the extracellular matrix *in vivo* [14,15]. HGF actions are mediated through binding to its specific cell surface receptor, Met. Activation of Met by HGF initiates various intracellular signal transduction cascades [16,17].

Met gene, located on chromosome 7q31, encodes a receptor protein tyrosine kinase (RPTK). Native Met protein is proteolytically processed to a heterodimer composed of a 50 kDa α subunit and a 145 kDa β subunit, linked together by disulfide bonds [18]. In addition to the intracellular tyrosine kinase domain, Met also has several other function domains including an extracellular ligand-binding semaphorin (SEMA) domain, a transmembrane domain, and a regulatory juxtamembrane (JM) domain [19,20]. Like other RPTKs, ligand binding induces Met dimerization and trans-phosphorylation of several tyrosines within the *C*-terminal domain of the β subunit, with concomitant activation of downstream effector molecules [15,21,22]. The function of Met is determined by these phosphorylated tyrosines, which serve as docking sites for signaling proteins [23,24]. Signaling proteins that are recruited to and activated in response to Met phosphorylation include: Akt, Cbl, Crk, cortactin, Erk, Fak, Gab1, Grb2, Jnk, paxillin, PI3 kinase, PLC-γ, Ras, Shc, Shp-2, Src, and STAT3 [15,25]. Among the tyrosines within the *C*-terminus, tyrosine residues 1234 and 1235, located within the catalytic domain, are required for tyrosine kinase activity [26,27]. Tyrosines 1349 and 1356 compose multifunctional docking sites for Src homology 2 (SH2) domain-containing molecules such as Gab1, Grab2, PI3 kinase, and Cbl. Phosphorylation of these two tyrosines is necessary and sufficient for transmission of down-stream signaling events [28,29,30]. For example, binding of Grb2 to tyrosine 1356 results in activation of Ras-Raf-MAP kinase pathway and promotes cell proliferation [31]. Recruitment of Gab1 and its associations with Shp-2 and PLC-γ results in HGF-induced branching morphogenetic activity [31,32].

## 3. Regulation of Met Signaling Pathway

### 3.1. Positive Regulation

CD44 is a cell surface adhesion molecule that is involved in cell-cell and cell-matrix interactions. A number of CD44 isoforms, which differ in their extracellular domain, can be generated via alternative splicing [33]. The extracellular domain of CD44v6, one of the CD44 isoform, participates in binding of HGF to Met and is required for activation of Met by HGF in epithelial cells [34]. In the absence of CD44v6, ICAM-1 can substitute for CD44v6 as a co-receptor for Met signaling, resulting in similar HGF-dependent c-Met pathway activation [35]. The cytoplasmic domain of CD44v6 is essential for efficient activation of Ras by promoting the assembly of signaling proteins including Grb2, SOS, F-actin, ezrin, radixin and moesin [36]. CD44v10, another CD44 isoform, has been reported to facilitate Met signaling in endothelial cells by providing structural and topographical support that facilitates HGF binding [37].

The extracellular domain of Met shares structural homology with plexins, which are transmembrane receptors for semaphorins. Semaphorins function to control axonal guidance in the nervous system. Plexins are also expressed in other tissues where Met is present [38]. The highly conserved cytoplasmic domain of plexins does not possess enzymatic activity, but is capable of interacting with small GTP-binding proteins to control cytoskeletal structures [39,40,41]. In the absence of HGF, Met can form complexes with plexins and can be stimulated by semaphorins, leading to activation of Met signaling pathway and biological responses [42,43].

Although α6β4 integrin does not possess an intrinsic catalytic activity, it has been shown to promote invasive growth. α6β4 integrin physically interacts with Met and Met activation by HGF leads to tyrosine phosphorylation of the β4-subunit cytoplasmic domain. The resultant phosphorylated tyrosines provide supplementary docking sites for the recruitment of PI3 kinase, Shc, and Shp2 [44,45]. For example, recruitment of adapter protein Shc to β4 subunit cytoplasmic domain results in activation of Ras-MAPK pathway [46]. Although interaction between α6β4 integrin and Met does not affect Met tyrosine kinase activity or Met C-terminal tyrosine phosphorylation, these additional docking sites on α6β4 integrin cooperate with Met to potentiate activation of downstream pathways to achieve full scale Met-dependent responses and magnify Met biological functions.

Several studies have demonstrated that Met can be trans-activated by other receptor protein tyrosine kinases (RTKs) [47]. For example, in the absence of HGF, ligand activation of EGFR can activate Met in cells that express both Met and EGFR [48]. Similarly, activation of RON RTK causes Met transactivation in a HGF-independent manner [49]. In addition, G-protein-coupled receptor agonists, such as bradykinin and thrombin, can induce Met activation via a reactive oxygen species-dependent mechanism [50], which likely involves inhibition of protein tyrosine phosphatase activity (see below).

### 3.2. Negative Regulations of Met

#### 3.2.1. Negative Regulation of Met by Protein Tyrosine Phosphatases (PTPs)

Met plays important roles in both physiological and pathological conditions. The function of Met is determined by phosphorylation of *C*-terminal tyrosines, which serve as docking sites for down-stream signaling molecules. Therefore, tyrosine phosphorylation is synonymous with Met activation. Net tyrosine phosphorylate of Met is determined by dynamic equilibrium of intrinsic tyrosine kinase activity of Met and counteracting protein tyrosine phosphatases (PTPs). Several PTPs have been shown to regulate Met tyrosine phosphorylation. Palka *et al*. has demonstrated that DEP-1 (also known as RPTP-J) preferentially dephosphorylates tyrosines 1349 and 1365. However, DEP-1 does not appear to affect Met-mediated MAP kinase activation [51]. Antisense knockdown of RPTP-LAR has been shown to increases tyrosine phosphorylation of Met [52]. PTP1B and TCPTP have also been shown to dephosphorylate tyrosines 1234/1235 of Met [53]. 

RPTP-β regulation of Met tyrosine phosphorylation and biological functions has been extensively studied. Purified RPTP-β directly dephosphorylates purified Met and substrate-trapping mutant of RPTP-β specifically interacts with Met in intact cells. In addition, expression of RPTP-β reduces both basal and HGF-induced Met phosphorylation at tyrosine 1356 and inhibits downstream MEK1/2 and Erk activation, while shRNA-mediated knockdown of RPTP-β increases basal and HGF-stimulated Met phosphorylation at tyrosine 1356 [54]. Furthermore, expression of RPTP-β in HNSCC cells decreased Met tyrosine phosphorylation, downstream signaling, and HGF-induced responses including cell proliferation, migration and invasion. Knockdown of RPTP-β in HNSCC cells enhanced these functions [55]. 

Normal epithelial cells express Met, but not HGF [19]. HGF is secreted by stromal cells to activate Met, in a paracrine fashion. Completion of an autocrine loop by expression of HGF in epithelial cells, or overexpression of Met leads to transformation [56,57]. Down-regulation of RPTP-β, with or without increased expression of Met, would be expected to result in activation of the Met pathway [55]. 

#### 3.2.2. Negative Regulation of Met by Receptor Endocytosis

Met internalization is a major negative regulatory mechanism for desensitizing Met signaling pathway. HGF-induced endocytosis of Met, terminates signaling by sequestering Met in lysosomes, where it is degradated and prevented from recycling to the plasma membrane. Tyrosine 1003, located in the juxtamembrane domain of ligand-activated Met, recruits E3 ubiquitin ligase Cbl, resulting in mono-ubiquitination of Met at multiple sites [58]. Ubiquitylated Met is then recognized by endocytic adaptors that contain ubiquitin-binding domains and targets Met to the endosomal network for sequestration and degradation [59,60].

#### 3.2.3. Other Negative Regulatory Mechanisms

Under steady-state conditions, Met is also subjected to low level of proteolytic cleavage independent of ligand or ubiquitination. The *N*-terminal extracellular domain is first cleaved by a disintegrin and metalloprotease (ADAM), resulting in a membrane-anchored cytoplasmic tail. The membrane-associated cytoplasmic region is further processed by γ-secretase mediated proteolysis into labile intracellular fragments for ultimate degradation by proteosomes [61].

In addition, LRIG1, a transmembrane leucine-rich repeat and immunoglobulin (Ig)-like domain-containing protein, interacts with Met and negatively affect Met protein stability in HGF and Cbl independent manner, thereby, negatively regulating Met function [62].

miRNAs are small noncoding endogenous RNAs that can negatively regulate protein expression by targeting transcripts for degradation and by blocking translation [63]. A number of miRNAs have been demonstrated to down-regulate MET protein expression, including miR-133b, miR-199*, miR-34b, miR-34c, miR-23b, and miR-198 [64,65,66,67].

## 4. The Role of Met in Cancer Progression and Metastasis

In addition to its key participation in many physiological processes, numerous *in vivo* and *in vitro* studies point toward the importance of Met in human malignancy. Stimulation of Met by HGF enhances cell proliferation, survival, dissociation, migration, morphogenesis, formation of blood vessels, and degradation of extracellular matrix, all characteristics that are associated with invasive cell phenotype [68]. Met pathway also plays key roles in epithelial-mesenchymal transition, which is involved in tumor invasion [11]. Many types of cancer exhibit sustained Met tyrosine phosphorylation, including carcinomas of the head and neck, breast, colon, kidney, liver, lung, ovary, prostate, thyroid, melanoma, and sarcoma [69,70,71].

Aberrant Met signaling can be achieved by Met or HGF gene over-expression, Met point mutations, amplification, or gene rearrangement, leading to constitutive tyrosine kinase activity. Met was originally isolated as TPR-Met oncogene, which possesses ligand-independent tyrosine kinase activity, due to chromosomal rearrangement of translocated promoter region (TPR) in chromosome 1 fused to Met *C*-terminal sequence in chromosome 7 [17,72]. This rearrangement was later confirmed in patients with gastric carcinoma [73].

Met point mutations have been identified in hereditary and sporadic papillary renal carcinoma, hepatocellular and gastric carcinomas and head and neck squamous cell carcinomas [74]. Large body of literature shows that aberrant regulation of HGF/Met signaling pathway is involved in many types of human cancers. Inappropriate expression of HGF/Met autocrine signaling leads to increased tumorigenesis and enhanced metastatic activity [57]. Met expression is strongly associated with tumor metastasis and correlates with poor prognosis [15,75,76].

Met can also be responsible for resistance of tumors against therapies. For example, in the presence of EGFR inhibitors, such as gefitinib, a subset of tumor cells with high Met expression (*i.e.*, with Met gene amplification) can escape EGFR inhibition. These cells become resistant to anti-EGFR therapy and can undergo rapid clonal expansion [77]. These findings have led to the proposal that EGFR and Met inhibitors should be used simultaneously to treat certain cancers [78,79,80].

## 5. Met Axis in Head and Neck Cancer

### 5.1. Over-Activation of Met Axis in HNSCC

Over-expression of Met has been reported in nearly all types of HNSCC, including cancer of the hypopharynx, larynx, and oral cavity. This over-expression often correlates with more advanced clinical stages especially the nodal stage [81,82,83,84]. Knowles and colleagues reported that approximately 80% of primary HNSCC tumors display abnormal expression of HGF, Met or both [85]. Cortesina *et al*. reported that Met was over-expressed in most HNSCC specimens they analyzed [86] and Galeazzi *et al*. reported that Met over-expression was detected in 70% of HNSCC samples they tested, even more so in lymph node metastases [87]. Expression of Met and/or its ligand HGF increases during progression of HNSCC and there is a substantial increase of Met levels in affected lymph nodes, compared to corresponding primary tumors [82,83,84,87,88]. High Met expression is significantly correlated with reduced patient survival rate because these patients are more likely to have local recurrences and are more likely to develop distant metastases [89]. Patients with higher Met expression also have inferior response to both radiotherapy and chemotherapy [90,91]. In addition, elevated serum levels of HGF are significantly associated with advanced tumor metastasis stage and poor survival in HNSCC [92]. Increased expression of HGF has also been positively linked to lymph node metastasis of HNSCC *in vivo* [81,82,93]. 

Consistent with *in vivo* data, HGF stimulation of Met-expressing HNSCC cell lines promotes an invasive phenotype [15,94]. Furthermore, activating mutations of Met are specifically selected during HNSCC metastasis [95]. Transcripts of these mutant alleles are highly represented in metastases, but barely detectable in primary tumors, suggesting that cells carrying these Met mutations have growth advantage and are selected during clonal expansion and metastatic spreading. Genetic transfer of mutant Met to HNSCC cells confers invasive phenotype [95]. Interestingly, RPTP-β, a major negative regulator of Met phosphorylation and function in HNSCC, is significantly down-regulated in metastatic tumors in comparison with primary tumors [55]. Taken together, there exists compelling evidence that points to the importance of Met axis in metastasis of HNSCC. 

### 5.2. Met in HNSCC Progression

HNSCC metastasis is a multi-stage process that includes cellular detachment, epithelial-mesenchymal transition, proteolytic degradation of the basement membrane, migration through extracellular matrix, resistance to apoptosis in a new environment, and formation of new blood vessels [74]. Activation of Met signaling pathway drives cancer cells to acquire an invasive growth phenotype and promotes each of the stages of HNSCC metastasis.

E-cadherin is an important cell adhesion molecule in epithelial cells and disruption of E-cadherin mediated cell-to-cell adhesion promotes detachment of cancer cells from their primary sites [96], which is the first step in tumor invasion process. Activation of Met by HGF in HNSCC cell lines decreases E-cadherin expression and induces E-cadherin translocation from the cell surface membrane to the cytoplasm [97]. HGF up-regulates transcription factor Snail via MAP kinase and Egr-1 signaling pathways in HNSCC cell lines [98]. HGF-induced Snail expression not only suppresses E-cadherin expression, but also promotes epithelial-mesenchymal transition, a process that allows epithelial cells to gain a fibroblast-like phenotype that is essential for tumor invasion [99,100,101].

Activation of Met by HGF in HNSCC cells leads to activation of Erk and Akt kinases, and Ets-related transcription factor E1AF activation, which in turn results in up-regulation of urokinase-type plasminogen activator and matrix metalloproteinases (MMP-1, 3 and 9) production [94,102]. These proteases are capable of degrading the extracellular matrix and may cooperate with MT-MMP-1 to facilitate cell migration through basement membrane, a critical step in tumor invasion. Met activation enhances HNSCC cell migration through mechanisms that involve Ras-related small G-protein Rho, and focal adhesion kinase [103,104]. Activation of Erk and Akt kinases by HGF promotes cell survival by protection against cell death that results from detachment from the extracellular matrix (anoikis) [105]. 

Higher serum HGF levels correlate with higher levels of angiogenic factors such as interleukin-8 (IL-8) and vascular endothelial growth factor (VEGF) in patients with HNSCC [106]. *In vitro* experiment showed that HGF treatment of HNSCC cells increased IL-8 and VEGF production [107]. Furthermore, HNSCC cells from tumors that are enriched in Met positive cells are able to form spherical colonies in anchorage-independent culture condition and are capable of self-renewal. These Met enriched cells are able to recapitulate the heterogeneity of the parental tumors *in vitro* and *in vivo*. This capability is further enhanced by the presence of CD44 which facilitates Met activation. In addition, these Met positive cells have enhanced metastatic ability and are resistant to cisplatin treatment [108]. 

### 5.3. Met in Resistance to HNSCC Therapy

EGFR is over-expressed in over 80% of HNSCC tumors [109] and anti-EGFR therapies using EGFR tyrosine kinase inhibitors (TKIs) or EGFR neutralizing antibodies have been used in combination with radiation therapy and chemotherapy against HNSCC [110]. Met signaling pathway induces invasive tumor growth and shares many components and mechanisms with EGFR signaling pathway [111]. Therefore, the Met axis may substitute and bypass EGFR functions when EGFR is inhibited by anti-EGFR therapies [112,113]. Indeed, over-activation of Met has been reported to correlate with resistance to EGFR neutralizing antibody cetuximab and other EGFR inhibitors in HNSCC cell lines [9]. Stabilization of Met in HNSCC cell lines by over-expression of cortactin, a key regulator of dynamic actin networks and modulator of receptors signaling, increases Met activity and resistance to EGFR tyrosine kinase inhibitor gefitinib [114]. Furthermore, low expression of Met is a predictive factor for a positive response in HNSCC patients treated with chemotherapy [91]. Ligand-independent activation of Met pathway by activated c-Src in HNSCC cells provides an alternate survival pathway and contributes to erlotinib resistance [115]. 

## 6. Targeting the MET Axis in HNSCC:

### 6.1. Cancer Drugs Targeting Met Pathway

In view of its high expression in various human malignancies and its involvement in mediating invasive growth of many types of cancers, Met has been identified as an attractive target for cancer treatment. Many types of inhibitors have been developed to inhibit this signaling pathway, including receptor antagonists, small-molecule tyrosine kinase inhibitors (TKIs), and monoclonal antibodies (mAbs). Among them, mAbs such as rilotumumab and onartuzumab, and TKIs such as tivantinib, crizotinib, foretinib and cabozantinib have recently been tested in phase I and II clinical trials [9]. 

NK4 is a receptor antagonist consisting of *N*-terminal 4 kringle domains of the α-chain of HGF. NK4 competes with HGF binding to Met, thereby inhibits HGF-dependent Met tyrosine phosphorylation, down-stream signal pathways, cell proliferation, and invasion [116,117,118]. In addition, NK4 inhibits VEGF and basic FGF induced angiogenesis independent of its HGF-antagonist function [117]. No clinical trial has been initiated with this anti-Met reagent.

Rilotumumab (also known as AMG102), is a humanized monoclonal antibody that selectively binds and neutralizes HGF, thereby preventing HGF binding to Met and interfering with Met activation [119,120]. An acceptable safety profile was shown in phase I and II clinical trials with mild side effects such as fatigue and gastrointestinal symptoms [121,122]. In advanced or metastatic gastric or gastroesophageal cancers, which have high Met protein expression, treatment with rilotumumab in combination with chemotherapy improves progression-free survival [123].

Onartuzumab is a humanized mono-valent anti-Met antibody (OA5D5/MetMAb). It binds Met with high affinity, thereby preventing HGF binding to Met and consequent Met phosphorylation, and activation of downstream signaling events. In preclinical studies, onartuzumab has been shown to inhibit tumor growth in multiple tumor models [124,125]. Onartuzumab is currently in a randomized, multi-center, double-blind human phase III clinical trial to evaluate its efficacy and safety in gastric cancer (ClinicalTrials.gov Identifier: NCT01662869).

Tivantinib (ARQ197) is a non-competitive, ATP-site selective, small molecule inhibitor of Met tyrosine kinase [126]. Phase I trials have shown a favorable safety profile and preliminary results suggest potential anti-invasive activity for this compound in advanced solid tumors [127]. Tivantinib is currently in phase II clinical trial.

Cabozantinib (formerly known as XL184) is another multi-kinase inhibitor against Met, VEGFR2, and Ret. Phase I and II studies have shown promising results for improvement on patient survival [128]. A randomized phase III trial of cabozantinib in thyroid cancer has been initiated (clinicaltrials.gov Identifier: NCT00704730). 

### 6.2. Targeting Met Axis in HNSCC Cells and HNSCC Patients

Met targeted therapy is a relatively new area of clinical investigation in HNSCC treatment. Met pathway inhibitors might be used in combination with chemotherapy, radiotherapy, or immunotherapy reagents to overcome potential tumor resistance and to improve overall clinical outcome. Several clinical trials are ongoing to investigate the effects of monoclonal antibodies and small molecular TKI against Met axis in patients with recurrent HNSCC.

Crizotinib (PF-02341066) is a potent and selective dual specific TKI for both Met and ALK. It exhibits inhibitory effects on Met-dependent proliferation, migration, and invasion of human tumor cells and is also effective against tumors with an activating ALK gene rearrangement [129]. In a preclinical HNSCC xenograft mouse model, crizotinib was shown to decrease tumor proliferation and increase apoptosis within the tumors, and delay HNSCC tumor growth by 50% [85]. Crizotinib has also been shown to suppress Met signaling, cell viability, and migration in HNSCC cells, and tumor angiogenesis in a HNSCC xenograft model [79]. Furthermore, combination treatment of crizotinib and EGFR inhibitor gefitinib disrupts cell proliferation and invasion significantly more than inhibition of each pathway alone in HNSCC cell lines [130]. These results suggest that anti-Met therapy has the potential to improve outcomes in HNSCC patients. 

Foretinib (formerly known as XL880) is a multi-targeted TKI whose primary targets are Met and VEGF [131]. It is the first orally bio-available small molecule targeting Met. A Phase II clinical trial of foretinib in HNSCC has been completed but the results have not been posted yet (clinicaltrials.gov Identifier: NCT00725764). LY2801653 is another orally bio-available multi-targeted TKI against Met and MST1R. It has been demonstrated to exhibit anti-tumor activities in mouse xenograft models in a preclinical study [132]. A Phase I Study of LY2801653 in patients with HNSCC is currently ongoing but no results are available (clinicaltrials.gov Identifier: NCT01285037).

## 7. Perspective and Conclusions

Since the discovery of Met more than 25 years ago, much has been learned about its roles in a broad spectrum of cellular and biological functions, including mitogenesis, morphogenesis, angiogenesis, migration, and invasiveness. While these biological processes are tightly regulated during embryogenesis, tissue homeostasis in adulthood, wound healing, and liver regeneration, aberrant activation of Met by gene amplification, gene re-arrangement, point mutations, protein over-expression, or loss of negative regulatory mechanisms can contribute to tumor initiation, progression, metastasis and resistance to therapy. Therefore, Met axis is an attractive target for cancer treatment and a number of Met pathway inhibitors have been developed and are currently in clinical trials. Anti-Met clinical studies for HNSCC are ongoing and results from these studies will shape the landscape for future use of Met inhibitors in HNSCC. Further clinical trials on the efficacy of Met inhibition in HNSCC and other types of solid tumors are warranted, given the important role of Met axis in HNSCC invasive growth.
